# Case of Amaurosis

**Published:** 1815-06

**Authors:** William John Crowfoot

**Affiliations:** Memb. of the Roy. Coll. of Surg. Beccles


*For the Medical and Physical Journal.*

### *Case of Amaurosis;* by Mr. Crowfoot.

IF the following case be thought worthy the notice of my professional brethren, you will please to give it insertion in your valuable Journal.

The symptoms and treatment previous to my seeing the patient, were communicated to me by Mr. Dashwood, and to that gentleman I owe the opportunity of the subsequent treatment.

S. L., aged 29, applied, on the 3d of February, for a pain on the top of the head, which, in a short time, had become so intolerable, as to render her incapable of attending to the duties of her family. She made no complaint of fever, nor could she attribute it to any blow or fall she had received. Her general health, which is at all times delicate, was not particularly impaired; nor was there any constitutional cause to which it could with propriety be assigned. She was the mother of one child, and, from the suppression of the menses for three months, might possibly be again pregnant. But this could hardly be the cause of her present sufferings.* Blisters upon the temples, and a bolus containing four grains of calomel, with a saline aperient draught, speedily and *completely* removed the pain. But she now spoke of imperfection in her sight, complained of a mist before her right eye, which gradually affected the left also; and, in the course of forty-eight hours, she was in a state of total darkness. Small doses of sulphat of zinc, with Cayenne pepper, were tried for a week or ten days, from which she thought she derived some benefit to her general health, but with no alleviation of the complaint. About the 13th I first saw the patient, with Mr. D. At this time she had no head-

---

* A few weeks after, these doubts were removed, by a regular return of the menstrual discharge.

ach, ach, nor did she describe any uncomfortable feeling (except the loss of sight before mentioned). The pupils were fully dilated, and quite insensible to the strongest light. The powerful agency of electricity promised to be most efficient; but we were by no means sanguine in its advantages. A cautious trial, however, was proposed and adopted. Sparks, at first slight, but gradually increased in number and intensity, were daily taken from both eyes; and this plan was uninterruptedly pursued for three weeks. After five or six days, she expressed with some confidence an amendment, without, however, being able to furnish us with any proofs of her progress. But, to use her own words, "there was a general sense of light, without being able to discriminate objects." At the end of a fortnight, she surprised us most agreeably by distinguishing some large letters painted upon the door, and so rapid was her improvement from this time, that it soon became unnecessary to persist in the remedy. I have only further to add, the patient continues well at this time, and is able to work with her needle by candle-light.

WILLIAM JOHN CROWFOOT,

*Memb. of the Roy. Coll. of Surg.*

*Beccles; April* 26, 1815.

